# Two-dimensional Nb_2_C MXene thin films for electro-ionic actuation

**DOI:** 10.1039/d6ra03119f

**Published:** 2026-07-10

**Authors:** Syed Sheraz Ali, Do Van Lam, Haleem Ud Din, Sheraz Ahmad, Raheel Abbas, Tawfik A. Saleh

**Affiliations:** a Interdisciplinary Research Center for Advanced Materials, King Fahd University of Petroleum and Minerals (KFUPM) Dhahran 31261 Kingdom of Saudi Arabia syed.ali.3@kfupm.edu.sa tawfik@kfupm.edu.sa; b Department of Mechanical Engineering, Korea Advanced Institute of Science and Technology (KAIST) Daejeon 34141 Republic of Korea; c Department of Physics Education, Chosun University Gwangju 61452 Republic of Korea; d Chemistry Department, King Fahd University of Petroleum and Minerals (KFUPM) Dhahran 31261 Kingdom of Saudi Arabia

## Abstract

Nb_2_CT_*x*_ MXene was synthesized through a Lewis acid-assisted molten salt etching process using silver chloride, providing a fluoride-free and safer alternative to conventional HF-based etching methods for converting the Nb_2_AlC MAX phase into layered Nb_2_CT_*x*_ sheets. Structural and surface analyses confirm the effective removal of the Al layer and the successful formation of conductive Nb_2_CT_*x*_, with preserved morphology and controlled surface terminations. The synthesized MXene was subsequently incorporated into a PEDOT:PSS (PP) matrix to fabricate flexible composite thin films for electromechanical actuation. Electrochemical characterization performed at various scan rates demonstrates stable capacitive behavior, delivering an areal capacitance of 206.62 mF cm^−2^. Compared with conventional PP films, the Nb_2_C–PP composite exhibits enhanced electroactive response and improved bending deformation, achieving a maximum displacement of 10.44 mm under low-amplitude alternating voltage input. To support the experimental findings, first-principles density functional theory (DFT) calculations were conducted to investigate the electronic structure and interfacial characteristics of Nb_2_CT_*x*_, providing insight into the improved charge transport and actuation performance. The combined experimental and theoretical results highlight molten salt-derived Nb_2_CT_*x*_ MXene as a scalable and efficient material platform for next-generation soft actuator applications.

## Abstract 2

Nb_2_CT_*x*_ MXene was synthesized by a fluoride-free molten-salt method using silver chloride as the etchant, providing a safer alternative to hydrofluoric acid (HF)-based methods for thin-film electroactive soft actuator applications.

## Graphical abstract

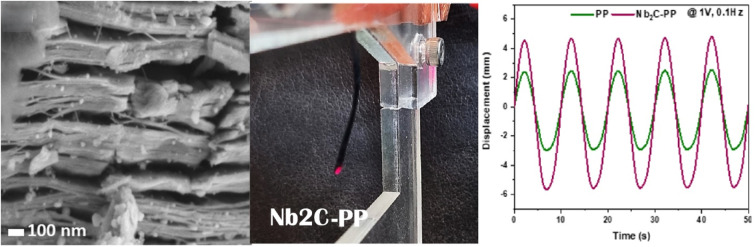

## 1. Introduction

The growing field of soft robotics has significantly reshaped the design of adaptive devices capable of safe interaction, large deformation, and biomimetic motion.^1–5^ Soft actuators, as the core driving components of these systems, have attracted considerable attention due to their ability to deliver controllable strain, fast response, lightweight operation, and mechanical compliance under low driving voltages.^6–8^ Their integration into flexible electronics, wearable systems, biomedical tools, and artificial muscle technologies has accelerated the demand for material platforms that combine high electrical conductivity with mechanical flexibility. Traditional polymers provide excellent deformability but often lack sufficient electrical performance, whereas metallic conductors offer superior conductivity but lack flexibility. To overcome this trade-off, advanced nanostructured materials such as graphene, carbon nanotubes, covalent organic frameworks, and, more recently, MXenes have emerged as promising candidates.^9–13^ Among these, MXenes are particularly attractive due to their layered structure, tunable surface chemistry, high conductivity, and ability to form flexible films or polymer composites.^14^ These characteristics position MXenes as highly promising materials for next-generation soft actuators where electrical transport, mechanical compliance, and structural stability must coexist within a single functional system.^15–18^

MXenes, a rapidly growing class of two-dimensional transition metal carbides and nitrides generated from layered MAX phase precursors, were first introduced in 2011. These materials are typically represented by the formula M_*n*+1_X_*n*_T_*x*_, where M denotes an early transition metal, X corresponds to carbon or nitrogen, and T_*x*_ represents surface termination groups that strongly influence their physicochemical behavior.^19–22^ Upon selective elimination of the A-layer from M_*n*+1_AX_*n*_ MAX phases, the three-dimensional layered solid transforms into a two-dimensional structure made up of metallic transition metal layers that contain carbon or nitride sheets.^23–27^ Owing to their metallic-level electrical conductivity, tunable surface chemistry, and hydrophilic character, MXenes can be processed into films, coatings, and composites with relative ease.^28–32^ Their layered architecture and accessible surface functionalities make them particularly attractive for applications requiring efficient charge transport and interfacial interactions,^33–35^ including sensing platforms, electromagnetic shielding, wearable systems, and, more recently, soft robotic components and electromechanical actuators.^36–39^

For the synthesis of MXenes from MAX precursor, several methods have been reported, including hydrofluoric acid (HF) etching, the Minimally Intensive Layer Delamination (MILD) strategy, direct HF-assisted routes, chemical vapor deposition (CVD), and, more recently, molten salt-based techniques.^40–44^ Although these methods successfully generate layered MXene structures, concerns regarding operational safety, environmental impact, and the use of corrosive fluoride-containing reagents remain significant. Unlike conventional HF-based approaches that naturally yield mixed surface terminations, molten salt etching enables more controllable and uniform surface functionalization depending on the conditions of the reaction.^45,46^ Molten salt etching has become a particularly appealing alternative from the perspectives of sustainability and decreased chemical hazards because it avoids fluoride chemistry and offers a relatively safer synthetic pathway.^47,48^ Other reported techniques, including CVD, alkali-assisted etching, and electrochemical methods, frequently involve aggressive reaction environments, relatively low production throughput, elevated processing costs, and reduced overall yields under demanding conditions.^49–51^ While both conventional and molten salt approaches have demonstrated scalability to varying extents, traditional fluoride-based methods are often limited by hazardous working conditions, increased production expenses, and complications during scale-up.^52–55^

Molten salt synthesis has also shown strong performance across diverse electrochemical energy storage and conversion systems, such as batteries, catalytic electrodes, and supercapacitors. Compared with conventional MXene synthesis routes that typically require extended reaction durations ranging from 24 to 176 h, molten salt etching can be completed within significantly shorter times, with reports indicating reaction periods as brief as 8 h.^56,57^ In addition to reduced processing time, molten salt-derived MXenes have demonstrated enhanced electrical conductivity relative to those obtained through conventional fluoride-based procedures.^58,59^ Furthermore, structural characterization shows that, contrary to the mixed terminations commonly observed in molten salt synthesis, more consistent and controllable surface terminations can be synthesized in HF-, LiF/HCl-, NaOH/KOH-based, or electrochemically etched MXenes.^60^ This improved surface regulation provides a distinct advantage in tailoring interfacial and electronic properties.^61^ Consequently, molten salt etching is increasingly recognized for its versatility, improved safety profile, scalability, and efficiency in controlling surface functionality when compared with conventional techniques.^62–69^ Numerous investigations have demonstrated successful synthesis and delamination of MXenes using molten salt strategies, with reported electrical conductivities reaching approximately 8000 S cm^−1^.^70^ Furthermore, S. Ali *et al.* reported the fabrication of cobalt-containing Ti_3_C_2_ MXenes *via* a molten salt route. They explored their applicability in soft robotic systems, highlighting the expanding potential of molten salt–engineered MXenes in responsive electromechanical devices.^71^ Recent studies have demonstrated MXene-based flexible actuators with programmable multidimensional deformation using MXene/polymer hybrid architectures. For example, MXene-CNF/PDMS actuators fabricated through electrohydrodynamic printing achieved a curvature of 2.34 cm^−1^ under near-infrared irradiation, showing improved actuation performance compared to conventional MXene/polymer systems.^72^

While conventional Ti_3_C_2_-based MXenes have been extensively investigated for electrochemical and actuation applications, Nb-based MXenes remain comparatively less explored, particularly for the application of soft electromechanical systems. Additionally, most previous studies rely on fluoride-based etching routes, typically involving hydrofluoric acid, or the MILD method, raising safety and handling concerns. Therefore, in this work, a molten salt-derived Nb_2_C MXene is utilized as an electroactive component in a soft actuator configuration, providing a safe acid-free synthesis pathway combined with functional actuation performance.

**Fig. 1:**
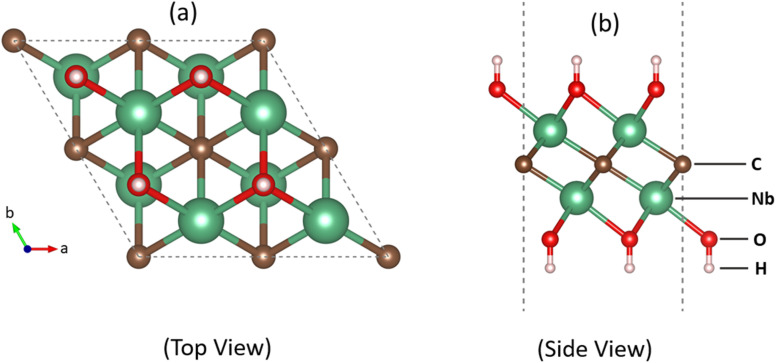
Functionalized structure of Nb_2_CT_*x*_ MXene, (a) top view, (b) side view.

In this work, Nb_2_CT_*x*_ MXene, as shown in Fig. 1, was prepared from Nb_2_AlC using a Lewis acid molten salt method, where silver chloride was used as the etching agent under acid-free conditions. The obtained Nb_2_CT_*x*_ was mixed with PEDOT:PSS (PP) to form flexible composite films for soft actuation. The Nb_2_C–PP actuator showed better bending performance than the pure PP film, reaching a peak-to-peak displacement of 10.44 mm at 1 V and 0.1 Hz, while the PP actuator showed 5.505 mm under the same conditions. The actuation response was also tested at low input voltages to evaluate its stability and movement behavior. In addition, first-principles density functional theory (DFT) calculations were carried out to understand the electronic properties of Nb_2_CT_*x*_ and to support the experimental results. These results show that molten salt-prepared Nb_2_CT_*x*_ can improve the actuation performance of polymer-based soft actuators.

## 2. Material and methods

### 2.1. Chemicals

Niobium aluminum carbide (Nb_2_AlC) MAX phase and silver chloride (AgCl, 99.9%) were purchased from Sigma-Aldrich (USA). Sodium chloride (NaCl, 99.0%) and potassium chloride (KCl, 99.0%) were also obtained from Sigma-Aldrich. *N*,*N*-Dimethylacetamide (DMAc, 99.8%) and dimethylformamide (DMF) were used as received. Nafion solution and 1-ethyl-3-methylimidazolium tetrafluoroborate (EMIMBF_4_, 98%) were used to prepare the membrane. Poly(3,4-ethylenedioxythiophene):poly(styrene sulfonate) (PEDOT:PSS; CLEVIOS™ PH 1000) was supplied by Heraeus (Germany). All chemicals were used as received without further purification.

### 2.2. Synthesis of Nb_2_CT_*x*_ MXene

Nb_2_CT_*x*_ MXene was prepared using a Lewis acid-assisted molten salt etching method. Initially, 1 g of powdered Nb_2_AlC MAX was weighed. Three grams of silver chloride (AgCl) were measured separately. Using a mortar and pestle, the powders were combined and crushed for ten minutes. After that, 0.75 g of NaCl and 0.81 g of KCl were added to the mixture and ground thoroughly to obtain a uniform blend. The mixed powder was transferred into an alumina boat and placed inside a tube furnace under continuous argon flow. The furnace temperature was raised to 750 °C and maintained for 7 h. After the heat treatment, the furnace was allowed to cool naturally to room temperature under an argon atmosphere. The obtained product was washed repeatedly with deionized (DI) water to remove residual salts. Centrifugation was carried out at 9000 rpm for 15 min per cycle, and the washing process was repeated ten times. The final product was vacuum filtered using deionized water and dried overnight under vacuum to obtain Nb_2_CT_*x*_ MXene powder.

### 2.3. Preparation of EMIMBF_4_-Embedded Nafion membrane

For membrane preparation, 1 g of Nafion was added to 40 mL of dimethylacetamide (DMAc) and stirred at 60 °C until completely dissolved. The solution was stirred for 24 h to ensure uniform mixing. After complete dissolution, 0.62 g of EMIMBF_4_ was added, and the mixture was stirred at 45 °C for another 24 h. Then, 5 mL of the prepared solution was poured into a Petri dish and dried in a vacuum oven at 90 °C for 7 h to obtain a flexible ionic Nafion membrane. The estimated Nafion membrane thickness falls within the commonly reported range for Nafion-based soft actuators, with a typical thickness of approximately 120 µm.^1^

### 2.4. Fabrication of Nb_2_CT_*x*_ – PEDOT:PSS (PP) soft actuator

To prepare the electrode material, Nb_2_CT_*x*_ powder was dispersed in 1 mL of DMF and sonicated to obtain a uniform suspension. This dispersion was mixed with PEDOT:PSS (PP) solution (0.5 mg mL^−1^) to form the active electrode mixture. The prepared mixture was drop-casted as 1 mL onto one side of the EMIMBF_4_-embedded Nafion membrane and dried at 65 °C for 50 min. After drying, the membrane was flipped, and the same coating procedure was repeated on the other side to form a symmetric electrode structure. Finally, for electromechanical actuation analysis, the fabricated Nb_2_CPP actuator films were cut into dimensions of approximately 3 cm × 1 cm, with a thickness of 140 µm.

### 2.5. Material characterization

The structural properties of the synthesized Nb_2_CT_*x*_ MXene were examined using X-ray diffraction (XRD) with a SmartLab RIGAKU powder diffractometer operated with a step size of 0.02°. The surface morphology and microstructure were observed using a field emission scanning electron microscope (FE-SEM, JSM-IT800, JEOL). Transmission electron microscopy (TEM) analysis was carried out using a Thermo Fisher field emission TEM operating at 200 kV. Fourier transform infrared (FTIR) spectra were recorded using a Nicolet iS50 spectrometer (Thermo Fisher Scientific) in KBr pellet mode to study surface functional groups. The surface composition and chemical states of the elements were analyzed using X-ray photoelectron spectroscopy (XPS, Nexsa G2, Thermo Scientific).

### 2.6. Electrochemical characterization

A VeraStat3 electrochemical workstation (Princeton Applied Research) was used to conduct electrochemical measurements. Ag/AgCl was used as the reference electrode, platinum wire was used as the counter electrode, and the produced Nb_2_CT_*x*_ electrode was used as the working electrode in a typical three-electrode setup. The electrolyte used was a 1 M solution of sulfuric acid (H_2_SO_4_). The active substance was combined with polytetrafluoroethylene (PTFE) and acetylene black in an 85 : 10 : 5 weight ratio to make the electrode. After adding a small amount of isopropyl alcohol to create a homogenous paste, the mixture was sonicated for half an hour. Before testing, the mixture was applied to a glassy carbon electrode and vacuum-dried for at least three hours at 50 °C.

### 2.7. Soft actuator measurement

The electromechanical performance of the soft actuator was evaluated using a National Instruments data acquisition system (NI-PXI1042Q with NI-PXI 6252 board). A current amplifier (UPM1504) was used to supply electrical signals, and the bending displacement was recorded using a laser displacement sensor (LK031, Keyence).

### 2.8. Computational analysis

Density functional theory (DFT) calculations were carried out with the use Vienna *Ab initio* Simulation Package (VASP) approach, which was employed to calculate the electronic band structure and density of states (DOS).^73^ The exchange–correlation interactions were described using the Perdew–Burke–Ernzerhof (PBE) formulation of the generalized gradient approximation (GGA).^74^ A plane-wave basis set with projector augmented-wave (PAW) potentials was employed.^75^

## 3. Results and discussion

In the Nb_2_CPP actuator, the applied electric field drives the migration of cations and anions within the EMIMBF_4_/Nafion electrolyte system toward oppositely charged electrodes, resulting in asymmetric ion accumulation and internal stress generation across the actuator thickness. The layered two-dimensional Nb_2_CT_*x*_ structure improves both electrical conductivity and interfacial charge storage capability, enabling more efficient charge distribution and ion transport within the PEDOT:PSS matrix. As higher capacitance allows greater charge accumulation at the electrode interface, the resulting electric field enhances ion mobility and promotes larger bending deformation. Therefore, the improved actuation behavior is attributed to the combined effect of enhanced conductivity, capacitive charge storage, and interfacial ion migration introduced by the Nb_2_CT_*x*_ layers.^71^

**Fig. 2:**
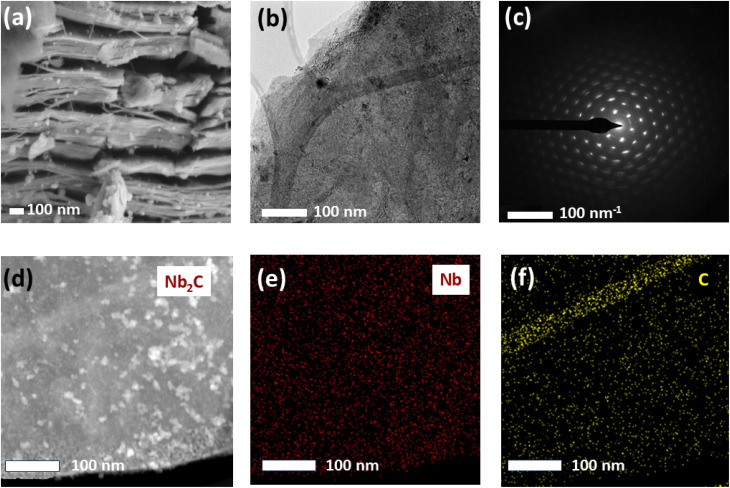
(a) SEM image showing Nb_2_CT_*x*_ MXene; (b) TEM image of Nb_2_CT_*x*_ MXene in resolution of 100 nm; (c) SAED pattern showing structure of Nb_2_CT_*x*_ MXene; (d–f) EDX spectra of Nb_2_CT_*x*_ MXene.

The morphology and microstructure of the synthesized Nb_2_CT_*x*_ were examined using SEM, TEM, and EDX analyses. The SEM image in Fig. 2(a) reveals a typical layered and partially delaminated structure characteristic of MXene materials obtained after molten salt etching. The sheets appear stacked with an expanded interlayer spacing, indicating successful removal of the Al layer from Nb_2_AlC and formation of Nb_2_CT_*x*_. The etched structure exhibits an accordion-like morphology, which is commonly observed in delaminated MXenes.

In Fig. 2(b), TEM analysis confirms the thin, flake-like sheet structure of Nb_2_CT_*x*_ MXene, demonstrating the presence of few-layer nanosheets. The selected area electron diffraction (SAED) pattern as shown in Fig. 2(c), displays well-defined diffraction rings, indicating that the crystalline nature of the carbide framework is retained after etching. Additionally, EDX analysis confirms the elemental composition of the material as shown in Fig. 2(d–f), showing the presence of Nb and C without detectable Al, verifying the successful conversion of the MAX phase into Nb_2_CT_*x*_ MXene.

**Fig. 3:**
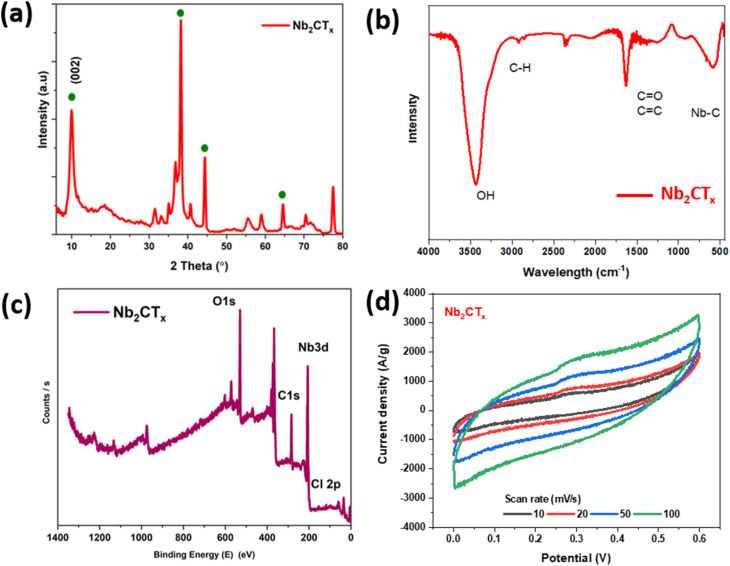
(a) X-ray diffraction pattern of Nb_2_CT_*x*_; (b) FTIR spectrum; (c) XPS survey spectrum of Nb_2_CT_*x*_; (d) cyclic voltammetry curves of Nb_2_CT_*x*_ recorded at different scan rates ranging from 10 to 100 mV s^−1^.

The structural transformation from Nb_2_AlC MAX phase to Nb_2_CT_*x*_ MXene was examined using X-ray diffraction. For the molten salt etched Nb_2_CT_*x*_ sample, the XRD shown in Fig. 3(a) confirms the formation of Nb_2_CT_*x*_ MXene.^76^ The (002) peak at 9.95° is accompanied by peak broadening. This shift toward lower 2*θ* values suggests an increase in the interlayer spacing due to the etching process and the formation of surface terminations. The reduction of higher-angle MAX reflections further confirms the successful conversion of Nb_2_AlC into Nb_2_CT_*x*_ MXene. The absence of additional impurity peaks indicates that no secondary crystalline phases were formed during the molten salt treatment. These observations verify the successful synthesis of layered Nb_2_CT_*x*_ MXene.

In Fig. 3(b), the Fourier transform infrared (FTIR) spectroscopy analysis confirms the formation of Nb_2_CT_*x*_ MXene and its characteristic surface vibrations.^77^ A broad absorption band centered around 3430 cm^−1^ corresponds to the stretching vibration of hydroxyl groups, indicating the presence of surface –OH terminations and adsorbed moisture. The peak observed near 1630 cm^−1^ is attributed to the bending vibration of interlayer water molecules. A prominent band around 1100 cm^−1^ is associated with C–O and Nb–O surface functional groups generated during the molten salt etching process. In the lower wavenumber region below 800 cm^−1^, the peaks correspond to Nb–C lattice vibrations, confirming that the carbide structure remains preserved after etching. These results verify the successful formation of Nb_2_CT_*x*_ with typical surface terminations of MXene materials.

To further analyze the surface chemical composition of Nb_2_CT_*x*_, X-ray photoelectron spectroscopy (XPS) was performed, and the survey spectrum is shown in Fig. 3(c). The spectrum confirms the presence of Nb, C, O, and Cl elements.^78^ The Nb 3d peak located at 206 eV corresponds to niobium bonded within the carbide framework. The C 1s peak observed at 284 eV confirms the Nb–C structure, while the O 1s peak at 530 eV indicates oxygen-containing surface terminations formed during the etching and washing processes. In addition, a Cl 2p peak centered at 198.4 eV was detected, confirming the presence of chloride surface terminations introduced during the molten salt etching. The atomic concentration of chlorine was approximately 0.79%, indicating a minor but detectable contribution of Cl on the MXene surface. The absence of any Al-related peaks further verifies the successful removal of the Al layer from Nb_2_AlC and the formation of Nb_2_CT_*x*_. The high-resolution XPS spectra for elements have been added in Fig. S1–S4, to identify the dominant terminations.

The electrochemical behavior of Nb_2_CT_*x*_ was investigated using cyclic voltammetry in a three-electrode configuration with KOH electrolyte, as shown in Fig. 3(d). The measurements were performed within a potential window of 0 to 0.5 V at scan rates ranging from 10 to 200 mV s^−1^. The CV curves display a quasi-rectangular shape across all scan rates, indicating typical capacitive behavior of the Nb_2_CT_*x*_ electrode. As the scan rate increases from 10 to 100 mV s^−1^, the current response increases proportionally while the overall curve profile remains largely preserved. This behavior suggests efficient charge transport and good rate capability of the material. The near-symmetric forward and reverse scans indicate stable and reversible electrochemical characteristics.

Although a slight distortion and tilt of the curves can be observed at higher scan rates, the overall shape remains consistent, demonstrating favorable ion diffusion and fast charge–discharge kinetics. The calculated areal capacitance of Nb_2_CT_*x*_ was found to be 206.62 mF cm^−2^, confirming its strong electroactive performance and suitability for electroactive actuator applications.

**Fig. 4:**
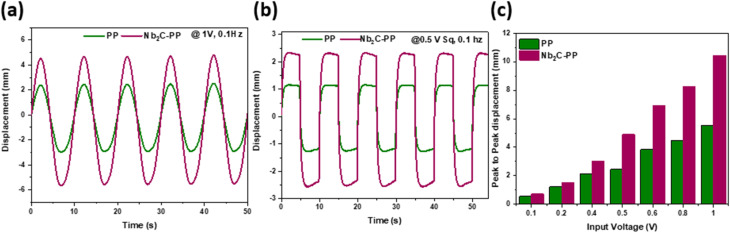
Electroactive soft actuator performance of Nb_2_C–PP compared with pristine PP. (a) Bending displacement under a sinusoidal input of ±1.0 V at 0.1 Hz; (b) displacement under a square wave input of ±0.5 V at 0.1 Hz; (c) peak-to-peak displacement of Nb_2_C–PP and PP at different sinusoidal input voltages ranging from 0.1 to 1.0 V at a fixed frequency of 0.1 Hz.

Under ambient conditions, the Nb_2_C–PP soft actuator's electromechanical response was evaluated and compared with the pristine PEDOT:PSS (PP) actuator. As seen in Fig. 4, a laser displacement sensor was used to measure the bending displacement under sinusoidal and square wave inputs at a fixed frequency of 0.1 Hz. The Nb_2_C–PP actuator was further demonstrated in Movie S1, for adaptive display and soft signaling applications under different electrical excitation frequencies.

As shown in Fig. 4(a), the Nb_2_C–PP actuator demonstrated a maximum peak-to-peak displacement of 10.4 mm at an applied voltage of 1 V and 0.1 Hz. The pristine PP actuator displayed a displacement of 5.50 mm under the identical testing conditions. The findings simply show that adding Nb_2_CT_*x*_ MXene greatly enhances the polymer-based actuator's actuation capability.

As seen in Fig. 4(b), a peak-to-peak displacement of 5.02 mm was measured when the Nb_2_C–PP actuator was driven by a square wave input of 0.5 V at 0.1 Hz. The pristine PP actuator showed a displacement of 2.2 mm under the same excitation conditions. The Nb_2_C–PP actuator's significantly higher bending response demonstrates the benefits of adding Nb_2_CT_*x*_ MXene to the polymer matrix. In comparison to the pristine PP system, the conductive Nb_2_CT_*x*_ layers improve charge transport and allow ion mobility within the actuator structure, leading to increased deformation.

To further evaluate the response behavior, the actuators were tested over a voltage range from 0.1 V to 1 V at 0.1 Hz. The Nb_2_C–PP device consistently demonstrated higher bending displacement compared to the PP actuator across the entire voltage range, as evident in Fig. 4(c). The improved performance can be attributed to the conductive layered structure of Nb_2_CT_*x*_, which enhances charge transport and facilitates ion movement within the actuator structure, leading to greater bending deformation under electrical stimulation.

Table 1 compares the actuation performance of the current work of the Nb_2_CPP actuator with previously reported actuator data under different operating conditions.

**Table 1:** A comparison of different actuators' bending displacement and corresponding actuation performance

Material name	Input voltage conditions at 0.1 Hz	Peak to peak bending displacement	References
Th-SNG/PP	0.5 V	2.30 mm	79
Th-SNG/PP	1 V	4.50 mm	79
P/(5G–4Ag)	0.5 V	0.50 mm	80
3D G–CNT–Ni/PP	1 V	4.84 mm	11
CA-IL-GN	1 V	7.50 mm	81
TOBC-IL-G	1 V	7.50 mm	82
TOBC-IL-G	2 V	11 mm	82
IL/PU/PEDOT/PSS/Xyl	2 V	2.90 mm	83
CBC-IL	2 V (0.5 Hz)	4.0 mm	84
GM-NG	3 V	6.40 mm	85
TS/PEGDA:IL/PP	3 V	5.50 mm	86
HLrGOP	5 V	8.52 mm	87
IL-IPMC	10 V	11.10 mm	88
**Nb**_**2**_ **C–PP**	**1 V**	**10.4 mm**	**This work**

**Fig. 5:**
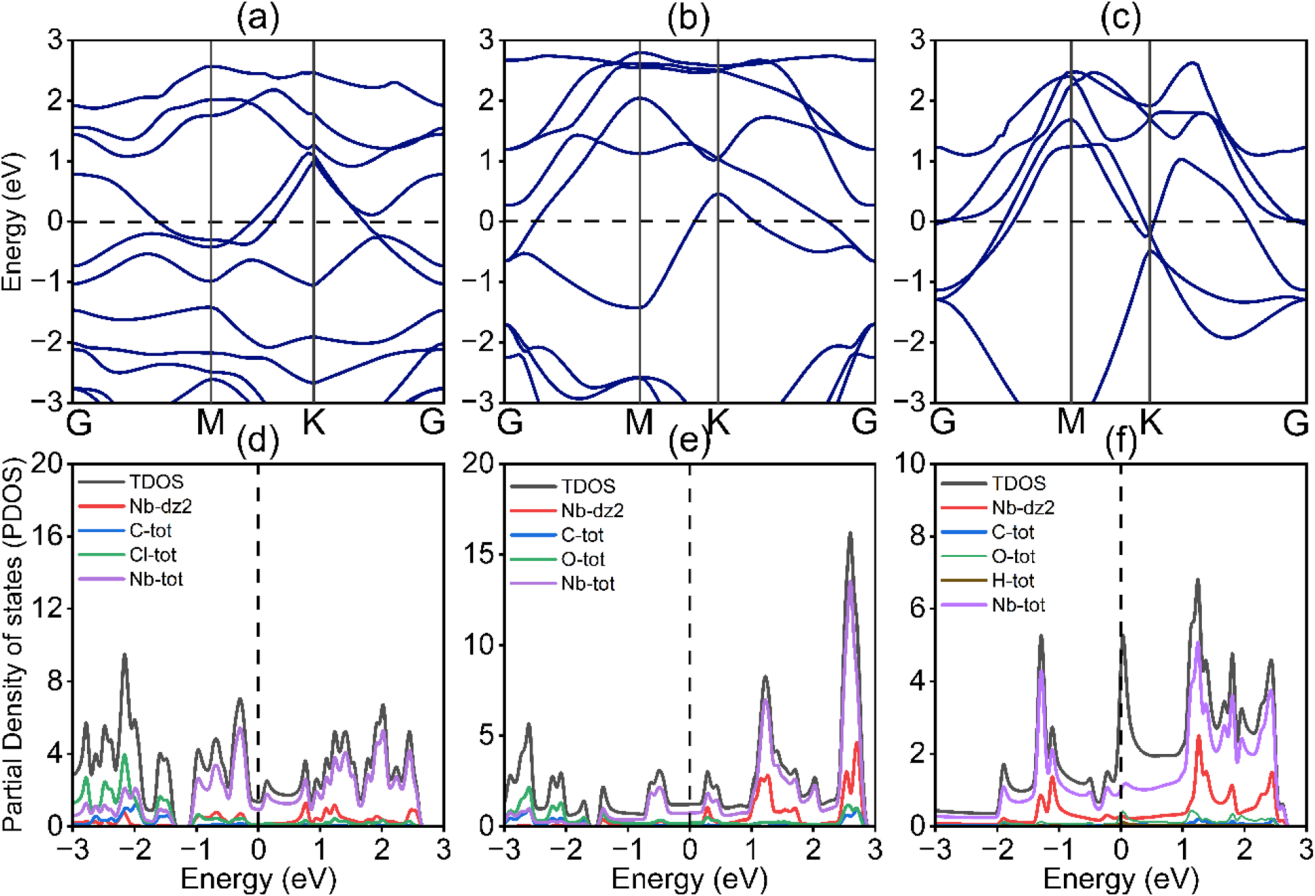
Electronic band structures of Nb_2_CT_*x*_ functionalized with (a) Cl, (b) O, and (c) OH terminations. The corresponding partial density of states (PDOS) are shown in panels (d–f), respectively. All functionalized structures exhibit metallic behavior, with bands crossing the Fermi level.

The optimized lattice parameters of the functionalized Nb_2_CT_*x*_ monolayer were found to be *a* = *b* = 3.32 Å. A vacuum spacing of *c* = 21 Å was introduced along the out-of-plane direction to prevent spurious interactions between periodic images, and van der Waals interactions were accounted for using the DFT-D3 dispersion correction method.^89^ The calculated bond lengths are 2.72 Å for Nb–C and 2.45 Å for Nb–Cl, while the Nb–C–Nb and C–Nb–Cl bond angles are 60.77° and 97.9°, respectively. These optimized structural parameters were used for subsequent band structure and partial density of states analyses. The electronic band structures of Nb_2_CT_*x*_ functionalized with (a) Cl, (b) O, and (c) OH terminations are presented in Fig. 5. In all cases, the calculated band structures reveal metallic behavior, as evidenced by the continuous crossing of bands at the Fermi level without the presence of a bandgap. To further analyze the electronic contributions near the Fermi level and clarify the origin of the metallicity, partial density of states (PDOS) calculations were performed. The PDOS results, shown in Fig. 5(d–f), indicate that the metallic character of the functionalized Nb_2_CT_*x*_ structures primarily originates from the Nb d_z^2^_ orbitals, which contribute significantly to the electronic states at the Fermi level. These findings confirm that surface functionalization with Cl, O, and OH does not disrupt the metallic nature of Nb_2_CT_*x*_, and that the Nb d-states dominate the electronic transport properties.

The improved actuation of Nb_2_CPP is attributed to the two-dimensional layered structure of Nb_2_CT_*x*_, which enhances electrical conductivity and provides efficient pathways for ion transport. Under applied voltage, cations and anions migrate within the Nafion membrane and accumulate at the electrode interfaces, generating asymmetric internal stress. The conductive Nb_2_C layers facilitate uniform charge distribution and faster ion movement, resulting in larger bending deformation. The capacitive behavior from CV measurements and DFT-supported electronic characteristics further confirms the enhanced electroactive response.

## 4. Conclusion

In summary, Nb_2_C MXene was successfully synthesized from Nb_2_AlC using a Lewis acid-assisted molten salt process with silver chloride as the etchant, providing a safe acid-free alternative to conventional HF-based MXene synthesis. Structural and surface analyses confirmed the successful formation of layered Nb_2_CT_*x*_ with preserved crystalline characteristics and surface terminations. The synthesized Nb_2_CT_*x*_ was incorporated into a PEDOT:PSS (PP) matrix to fabricate flexible Nb_2_C–PP thin films, which exhibited enhanced electromechanical performance compared to pristine polymer actuators under low applied voltages. Electrochemical measurements and DFT analysis further supported the improved charge transport and electroactive behavior of the composite system.

This work demonstrates the feasibility of utilizing non-titanium-based Nb_2_C MXene for electroactive soft actuator applications while extending MXene synthesis toward safer molten salt approaches without corrosive liquid acids. The development of a safe molten salt synthesis strategy broadens the pathway toward more sustainable production of MXenes and supports their future implementation in soft robotics, wearable electronics, and advanced energy storage technologies. Furthermore, continued exploration of different MXene compositions and hybrid thin-film architectures may further enhance multifunctional performance in next-generation smart material systems.

## Author contributions

Syed Sheraz Ali: writing – original draft, investigation, visualization, methodology, investigation, data curation, conceptualization. Writing – review & editing, Do Van Lam: conceptualization, investigation, data curation. Haleem Uddin: data curation, conceptualization. Sheraz Ahmad: data curation, conceptualization. Raheel Abbas: data curation. Tawfik A. Saleh: supervision, data curation, project administration, writing – review & editing. All authors discussed the results and commented on the paper.

## Conflicts of interest

There are no conflicts to declare.

## Supplementary Material

RA-016-D6RA03119F-s001

RA-016-D6RA03119F-s002

## Data Availability

Data will be made available on request. Supplementary information (SI) is available. See DOI: https://doi.org/10.1039/d6ra03119f.
