# Supplementary material for: Two-dimensional Nb2C MXene thin films for electro-ionic actuation
Source: RSC Adv. 2026 Jul 10;16(37):39512–22. doi: 10.1039/d6ra03119f (PMC13352260; doi:10.1039/d6ra03119f)
Supplement: Supplementary file 1 [file RA-016-D6RA03119F-s002.pdf]

Supplementary Information (SI) for RSC Advances.
This journal is © The Royal Society of Chemistry 2026

Supplementary Information

Two-Dimensional Nb_2C MXene Thin Films for Electro-Ionic Actuation

Syed Sheraz Ali[a*], Do Van Lam[b], Haleem Ud Din[c], Sheraz Ahmad[a], Raheel Abbas[b] and Tawfik A Saleh [a, d*]

[a] Interdisciplinary Research Center for Advanced Materials, King Fahd University of Petroleum and Minerals (KFUPM), Dhahran 31261, Kingdom of Saudi Arabia.

[b] Department of Mechanical Engineering, Korea Advanced Institute of Science and Technology (KAIST), Daejeon 34141, Republic of Korea.

[c] Department of Physics Education, Chosun University, Gwangju 61452, Republic of Korea.

[d] Chemistry Department, King Fahd University of Petroleum and Minerals (KFUPM), Dhahran 31261, Kingdom of Saudi Arabia

[*]**Corresponding Email: *syed.ali.3@kfupm.edu.sa, tawfik@kfupm.edu.sa**

Figures S1–S4 show the high-resolution XPS spectra of Nb_2CT_x, confirming the presence of characteristic Nb–C bonding along with typical surface terminations and minor residual species, thereby verifying the successful formation of the MXene structure.

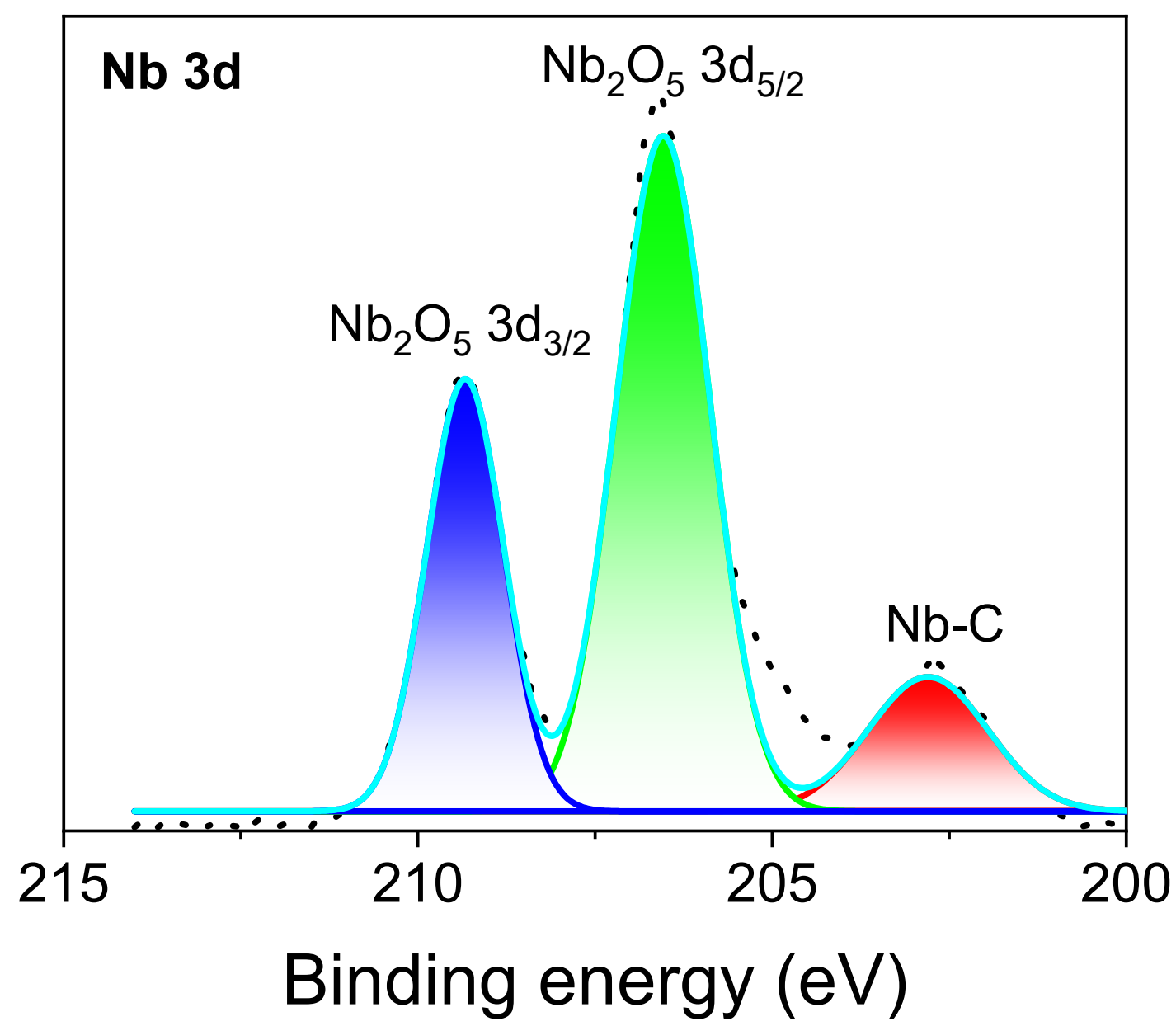

Fig S1 High-resolution XPS spectrum of the Nb 3d region for Nb_2CT_x MXene.

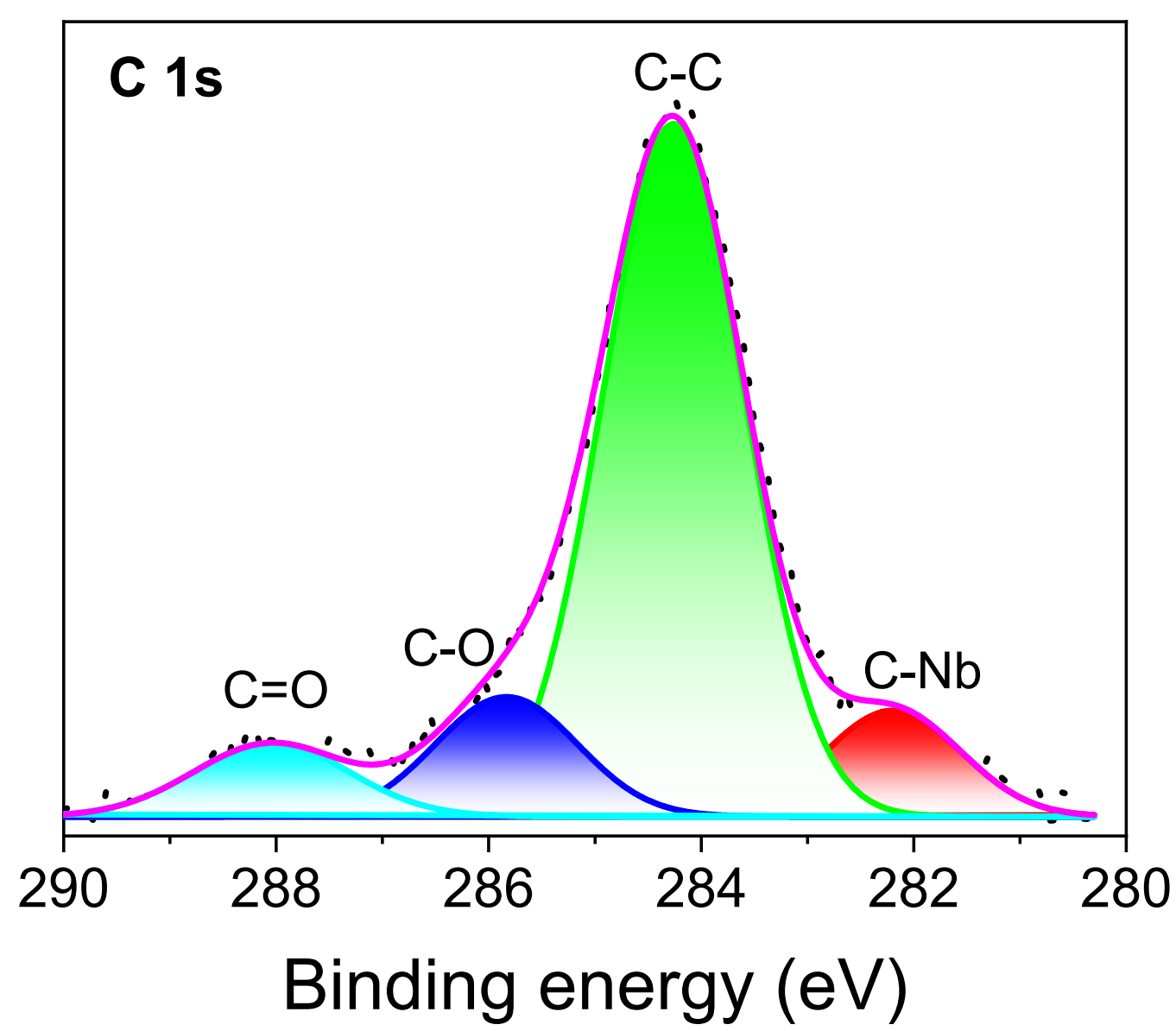

Fig S2 High-resolution XPS spectrum of the C 1s region for Nb_2CT_x MXene.

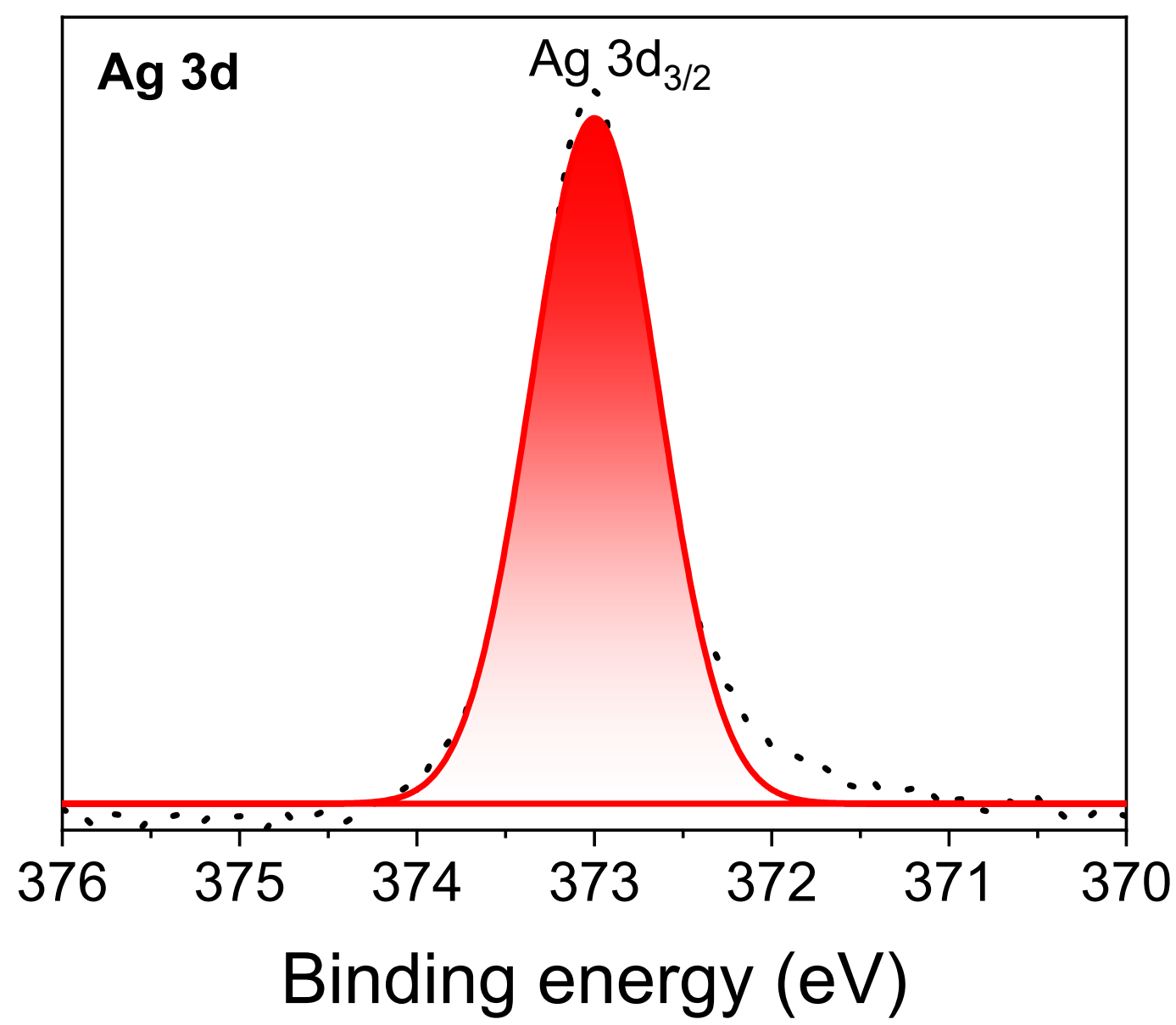

Fig S3 High-resolution XPS spectrum of the Ag 3d region for Nb_2CT_x MXene.

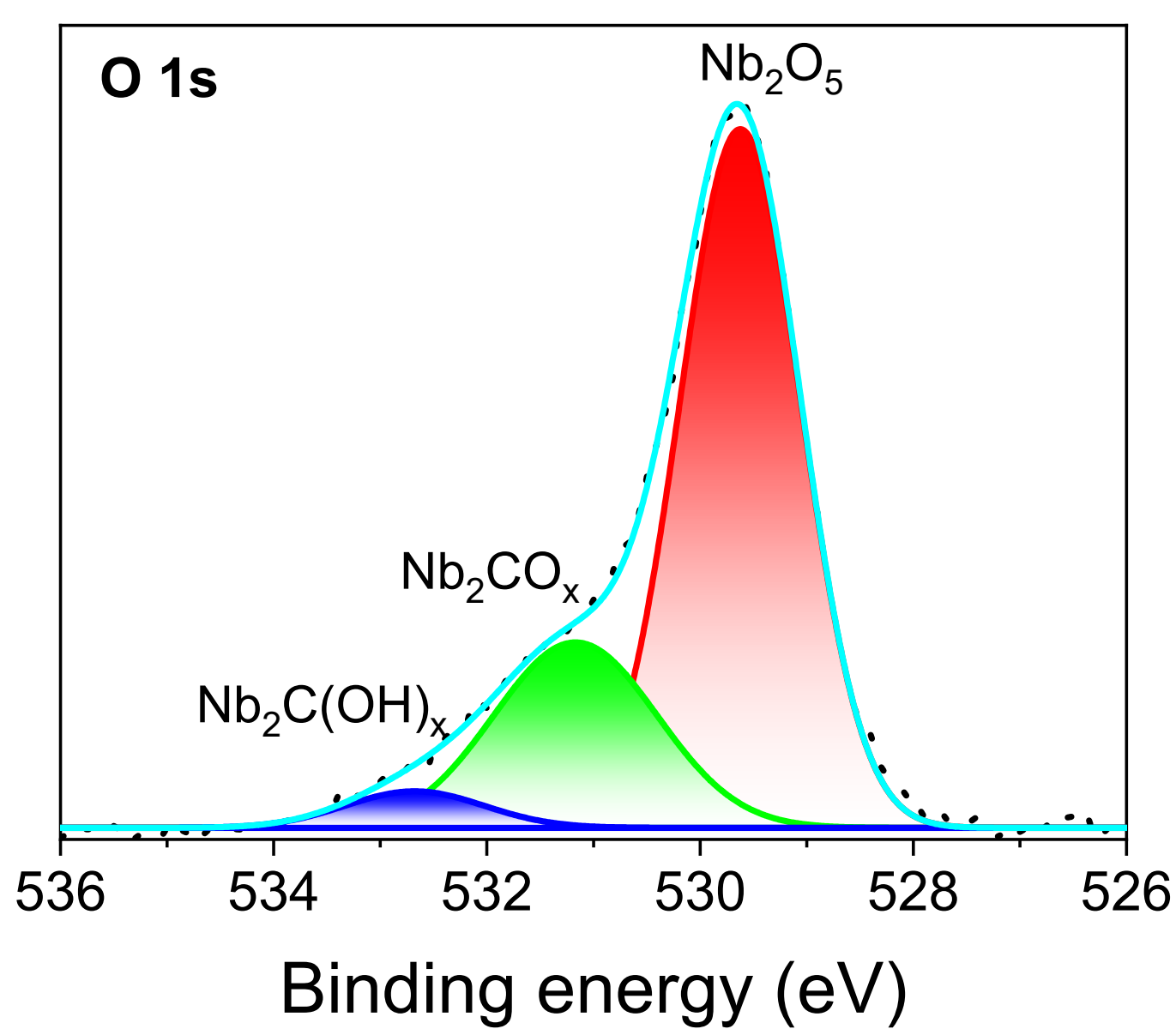

Fig S4 High-resolution XPS spectrum of the O 1s region for Nb_2CT_x MXene.

Table S1** **Binding energies, atomic concentrations, and corresponding elemental assignments obtained from XPS analysis of Nb_2CT_x MXene.

Peak	Binding Energy (eV)	Atomic %	Assignment
Ag 3d	373.01	4.90	Residual Ag species
C 1s	284.26	40.35	Carbon in Nb–C framework
Nb 3d	206.59	17.18	Nb–C bonding
O 1s	529.66	36.78	Oxygen-containing surface groups
Cl 2p	198.40	0.79	Chloride surface termination
